# Real-world cardiovascular safety profile of bexarotene for the treatment of mycosis fungoides

**DOI:** 10.1007/s10238-026-02282-5

**Published:** 2026-08-10

**Authors:** Charalampos Filippatos, Despina Fotiou, Peggy Kostakou, Maria Gavriatopoulou, Evangelos Terpos, Meletios-Athanasios Dimopoulos, Alexandros Briasoulis

**Affiliations:** 1 Department of Clinical Therapeutics, School of Medicine, National and Kapodistrian University of Athens, Athens, Greece; 2 Clinic & Laboratory of Pathophysiology, Department of Medicine, National and Kapodistrian University of Athens, Athens, Greece; 3 Department of Medicine, Korea University, Seoul, South Korea

**Keywords:** Bexarotene, Safety, Mycosis fungoides, Cutaneous T-cell lymphoma, Cardiovascular, Metabolic

## Abstract

Bexarotene has been established as an effective therapeutic agent for mycosis fungoides (MF), both in early, refractory disease and in advanced stages. It has a predictable, albeit significant, metabolic toxicity profile, raising concerns regarding long-term cardiovascular safety. We conducted a retrospective cohort study utilizing TriNetX to identify adults with MF. Patients were stratified by bexarotene exposure and 1:1 propensity score matched (PSM) for demographics, lifestyle factors, comorbidities, and baseline medications. After PSM, two well-balanced cohorts of 2,242 patients each were formed with a median follow-up of 4.0 years for both groups. Bexarotene treatment demonstrated no significant increase in the risk of the composite adapted-MACE endpoint (HR = 0.93, 95% CI: 0.78–1.11, *p* = 0.425). Similarly, no significant differences were observed for individual cardiovascular components, including acute myocardial infarction (HR = 0.95, *p* = 0.730), stroke (HR = 0.90, *p* = 0.514), or heart failure (HR = 0.93, *p* = 0.455). Conversely, bexarotene was associated with a significantly increased risk of metabolic and endocrine abnormalities, including hypertriglyceridemia (RR = 6.98, *p* < 0.001), hypothyroidism (RR = 4.93, *p* < 0.001), hyperlipidemia (RR = 2.18, *p* < 0.001), and hypercholesterolemia (RR = 1.99, *p* < 0.001). In the largest real-world cohort of MF patients treated with bexarotene to date, the substantial metabolic and endocrine abnormalities associated with treatment did not translate into an observed increase in cardiovascular events during a median follow-up of 4 years. These findings provide reassurance regarding the short- to intermediate-term cardiovascular profile of bexarotene when appropriate monitoring and management of lipid and thyroid abnormalities are maintained. However, longer follow-up is required to determine whether these metabolic effects influence long-term cardiovascular risk.

**Supplementary Information:**

The online version contains supplementary material available at 10.1007/s10238-026-02282-5.

## Introduction

Mycosis fungoides (MF) is the most prevalent form of the rare class of cutaneous T-cell lymphomas (CTCL) [1]. While disease progression to systemic stages is rare, when that occurs it requires a multidisciplinary approach [2, 3]. Bexarotene, a selective retinoid X receptor (RXR) agonist, has emerged as a cornerstone of the therapeutic arsenal for MF [4, 5]. Demonstrating significant efficacy and durable responses, it is one of the few approved drugs in this context [6]. However, its clinical utility is often complicated by a distinct and predictable profile of metabolic toxicities - most notably central hypothyroidism and profound hypertriglyceridemia [7–9].

While the endocrine and lipid-altering effects of bexarotene are well-documented in clinical practice [10, 11], their long-term impact on cardiovascular health remains a subject of significant concern [12]. In the general population, chronic hyperlipidemia and metabolic dysfunction are established drivers of atherosclerosis and major adverse cardiovascular events (MACE) [13, 14]. Yet, it remains unclear whether the drug-induced nature of bexarotene-associated metabolic derangements translates into a meaningful increase in cardiovascular morbidity or mortality in patients with MF.

Given the rarity of MF, large-scale real-world data evaluating “hard” cardiovascular endpoints in bexarotene-treated patients are lacking. To address this gap, we utilized the TriNetX global health research network to conduct the largest real-world cohort analysis of MF patients treated with bexarotene to date, aiming to determine whether exposure to the RXR agonist translates into an increased risk of cardiovascular complications.

## Methods

### Study design

The data used in this study were collected on June 4, 2026 from the TriNetX Global Health Research Network, which provided access to electronic medical records (diagnoses, procedures, medications, laboratory values, genomic information) from approximately 146 million patients from 111 healthcare organizations. Patients with MF were retrospectively enrolled in this study and were then stratified in separate cohorts, according to the presence or absence of treatment with bexarotene.

### Outcomes

The outcomes evaluated were: acute myocardial infarction (AMI, I21), cerebral infarction (Stroke, I63), heart failure (HF, I50), hypothyroidism (E03), hypertriglyceridemia (> 500 mg/dL triglycerides in serum, plasma or blood identified by the TNX curated lab code 9004), hypercholesterolemia (> 160 mg/dL LDL in serum or plasma identified by TNX curated lab code 9002) and hyperlipidemia (combination of hypertriglyceridemia and hypercholesterolemia). An adapted MACE outcome was formed, combining AMI, stroke, HF and coronary revascularization procedures, including percutaneous coronary intervention (PCI) and coronary artery bypass grafting (CABG) identified via current procedural terminology (CPT), healthcare common procedure coding system (HCPCS), and systematized nomenclature of medicine (SNOMED) codes [15]. New-onset use of antilipemic agents (VA CV350) and thyroid modifiers (VA HS851) was assessed among patients who were treatment-naïve for these specific drug classes during the 180-day baseline look-back window.

### Matching

To minimize selection bias and address potential confounding by indication, we performed 1:1 propensity score matching (PSM) using the TriNetX built-in analytics platform. PSM was performed based on covariates measured within a 180-day (6 months) look-back window prior to the index event. Patients were matched on: demographic and lifestyle factors (age at index, sex, race, ethnicity, and tobacco use or nicotine dependence), cardiovascular-related comorbidities (diabetes mellitus, disorders of lipoprotein metabolism, hypertensive diseases, ischemic heart diseases, cerebrovascular diseases, and atrial fibrillation or flutter), baseline medications (antilipemic agents, antihypertensives, insulin, other hypoglycemic agents, adrenal corticosteroids, antiplatelets, anticoagulants and thyroid modifiers), laboratory and vital signs (including BMI, systolic and diastolic blood pressure, hemoglobin A1c, HDL and LDL cholesterol, triglycerides, platelets and eGFR by CKD-EPI) and surrogates for tumor burden (lymphocytosis, eosinophilia, elevated lactate dehydrogenase, nodal involvement and Sezary disease/Erythroderma [16]). The quality of the matching was evaluated using standardized mean differences (SMD), where an SMD < 0.1 was considered to indicate perfect balance.

### Landmark analysis

To reduce potential immortal time bias arising from the variable period between initial MF diagnosis and systemic therapy, a landmark-based sensitivity approach was implemented. Upon granular evaluation of the treatment arm, a median duration of 249 days from diagnosis to bexarotene initiation was revealed, and a corresponding 249-day landmark window was enforced for the control group. Control patients were required to have at least 249 days of continuous post-diagnosis follow-up, with their index date established at the first clinical encounter immediately following this threshold. To ensure baseline risk comparability and prevent reverse causality, patients were excluded if they experienced a cardiovascular outcome of interest prior to their respective index dates.

### Statistical analysis

Baseline characteristics were summarized using medians with interquartile ranges (IQR) for continuous variables, and frequencies with percentages for categorical variables. Kaplan-Meier curves were used to estimate survival and time-to-event probabilities. Univariate Cox proportional hazards quantified the aforementioned estimates between groups, with Hazard Ratios (HR) and 95% Confidence Intervals (CI). The proportional hazards (PH) assumption was assessed for each outcome by the Schoenfeld residuals test within the TriNetX Analytics. Risk Ratios (RR) and Odds Ratios (OR) with 95% CIs were utilized to assess the likelihood between cohorts for the outcomes of 2.2. Regarding the management of missing data, no formal statistical imputation or complete-case elimination is performed by TriNetX’s built-in tools. Event counts and estimates are reported among evaluable patients per each outcome. All statistical analyses were performed within the TriNetX Analytics platform. Plots were recreated in R-Studio by utilizing extracted data. To account for multiple parallel hypothesis testing and control the inflation of the family-wise Type I error, a formal Bonferroni multiplier correction was applied to our secondary clinical and metabolic outcomes. With a primary significance threshold set at 0.05 for the pre-specified adapted-MACE endpoint, the adjusted significance threshold for our seven secondary individual cardiovascular and laboratory endpoints was established at 0.00714 (0.05 / 7). Secondary outcomes achieving a p-value below this corrected threshold were considered strictly statistically significant.

## Results

### Population characteristics and matching quality

A total of 2,495 MF patients treated with bexarotene were identified, with a median time to bexarotene initiation of 249 days. Furthermore, a total of 17,405 patients with no history of bexarotene treatment and at least 249 days of follow-up were also identified. After PSM for the prespecified covariates (Sect.  2.3), two well-balanced cohorts of 2,242 patients were formed (Supplementary Fig. 1). The median follow-up was 4.0 years for both cohorts.

The median age at index was 64 years in both cohorts, with a slight male predominance (57.5% vs. 58.6%) and a racial distribution primarily comprised of White individuals (65.1% vs. 65.5%). Clinical comorbidities were highly balanced across both groups, including hypertensive diseases (16.8% vs. 17.9%), disorders of lipoprotein metabolism (15.8% vs. 16.7%), and diabetes mellitus (8.0% vs. 8.8%). Cardiovascular risk factors, such as ischemic heart disease (4.2% vs. 4.1%), showed perfect balance.

Laboratory parameters and vital signs further demonstrated cohort parity; notably, patient subgroups per Systolic Blood Pressure, BMI, and Triglycerides were closely aligned. Baseline medications followed this trend, with comparable rates of adrenal corticosteroid use (38.8% vs. 38.3%) and antilipemic agent use (15.9% vs. 16.8%). Importantly, all included covariates achieved an SMD of less than 0.1, indicating that the cohorts were statistically comparable across all demographic, clinical, and laboratory dimensions prior to outcome analysis (Table 1, Supplementary Fig. 1).

**Table 1:** Baseline population characteristics

	Bexarotene (*n* = 2,242)	No bexarotene (*n* = 2,242)	SMD
Demographics			
Age (years)	64 (21)	64 (20)	0.006
White	1,449 (64.6%)	1,463 (65.3%)	0.013
Male	1,295 (57.8%)	1,283 (57.2%)	0.011
Black or African American	465 (20.7%)	481 (21.5%)	0.017
Not Hispanic or Latino	1,741 (77.7%)	1,773 (79.1%)	0.035
Comorbidities			
Hypertensive diseases	377 (16.8%)	386 (17.2%)	0.011
Disorders of lipoprotein metabolism and other lipidemias	352 (15.7%)	359 (16.0%)	0.009
Diabetes mellitus	180 (8.0%)	176 (7.9%)	0.009
Ischemic heart diseases	94 (4.2%)	86 (3.8%)	0.018
Atrial fibrillation and flutter	57 (2.5%)	69 (3.1%)	0.032
Cerebrovascular diseases	30 (1.3%)	35 (1.6%)	0.019
History of smoking	95 (4.3%)	103 (4.6%)	0.012
Tumor burden			
Lymphocytes > 4 × 10^3^/uL	133 (5.9%)	123 (5.5%)	0.019
Eosinophils > 0.7 × 10^3^/uL	125 (5.6%)	123 (5.5%)	0.004
LDH > 250 U/L	260 (11.6%)	259 (11.6%)	0.001
Nodal involvement	242 (10.8%)	250 (11.2%)	0.014
Sezary disease	117 (5.2%)	130 (5.8%)	0.025
Laboratory variables			
BMI (kg/m^2^)	28.2 (8.0)	28.3 (8.1)	0.011
TG (mg/dL)	128 (100)	126 (104)	0.011
HDL (mg/dL)	47 (20)	47 (19)	0.019
LDL (mg/dL)	100 (52)	99 (52.2)	0.019
HbA1c (%)	6.2 (1.4)	6.1 (1.7)	0.007
Systolic BP (mmHg)	132 (25)	131 (23)	0.072
Diastolic BP (mmHg)	76 (14)	75 (14)	0.096
PLT (U/L)	236 (97)	230 (90)	0.093
eGFR by CKD-EPI	83.7 (28.9)	83.6 (27.8)	0.008
Medications			
Adrenal corticosteroids	870 (38.8%)	872 (38.9%)	0.002
Antilipemic agents	365 (16.3%)	375 (16.7%)	0.012
Beta blockers	195 (8.7%)	205 (9.1%)	0.016
Angiotensin II inhibitors	132 (5.9%)	136 (6.1%)	0.008
Ace inhibitors	132 (5.9%)	127 (5.7%)	0.010
Oral hypoglycemic agents	107 (4.8%)	106 (4.7%)	0.002
Insulin	77 (3.4%)	70 (3.1%)	0.018
Antihypertensives	62 (2.8%)	68 (3.0%)	0.016
Thyroid modifiers	162 (7.2%)	173 (7.7%)	0.019
Antiplatelets	136 (6.1%)	134 (6.0%)	0.004
Anticoagulants	206 (9.2%)	216 (9.6%)	0.015

Note: All categorical variables are presented as count (percentage) and all numerical variables are presented as median (IQR); SMD= standardized mean differences, BP= blood pressure, BMI= body-mass index, LDH= lactate dehydrogenase, HDL= high-density lipoprotein, LDL = low-density lipoprotein, HbA1c = glycated hemoglobin, PLT= platelets, TG= triglycerides, eGFR= glomerular filtration rate

## Lipid and metabolic laboratory-related events

### Hypertriglyceridemia

A total of 352 patients on bexarotene developed severe hypertriglyceridemia (TGs > 500 mg/dL), compared to only 51 among those not treated with the drug, out of 2,135 and 2,160 evaluable, respectively. The cumulative risk was 16.5% and 2.4% between the two cohorts, respectively (RR = 6.98, 95% CI: 5.24–9.31, *p* < 0.001).

## Hypothyroidism

A total of 580 patients developed hypothyroidism while on bexarotene, compared to 166 among those not treated with the drug, out of 1,281 and 1,806 evaluable, respectively. The cumulative risk between the two groups was 45.3% and 9.2%, respectively, translating into a near 5-fold increase in the respective risks (RR = 4.93, 95% CI: 4.21–5.76, *p* < 0.001).

### Hypercholesterolemia

A total of 436 patients developed hypercholesterolemia while on bexarotene, compared to 211 who were not receiving the drug, out of 1,926 and 1,850 evaluable, respectively. The cumulative risk between the two cohorts was 22.6% and 11.4%, respectively. Bexarotene induced double the risk of hypercholesterolemia (RR = 1.99, 95% CI: 1.71–2.31, *p* < 0.001).

## Hyperlipidemia

Hyperlipidemia was observed in 714 patients on bexarotene and 318 controls, among 1,406 and 1,365 evaluable individuals, respectively. The cumulative risk was 50.8% and 23.3%, respectively. Those on bexarotene demonstrated a 2-fold increase in crude risk (RR = 2.18, 95% CI: 1.95–2.43, *p* < 0.001).

For all of the aforementioned lipid and metabolic laboratory-related events, time-to-event analysis demonstrated a significantly shorter time to onset in the bexarotene cohort (all Log-rank *p* < 0.001; Supplementary Figs. 2–5). This excess risk is observed within the first year following treatment initiation, after which the event rates between the two groups tend to stabilize. As this pattern results in a violation of the PH assumption (Schoenfeld residuals test, all *p* < 0.001), no hazard ratio estimates are reported.

**Fig. 1:**
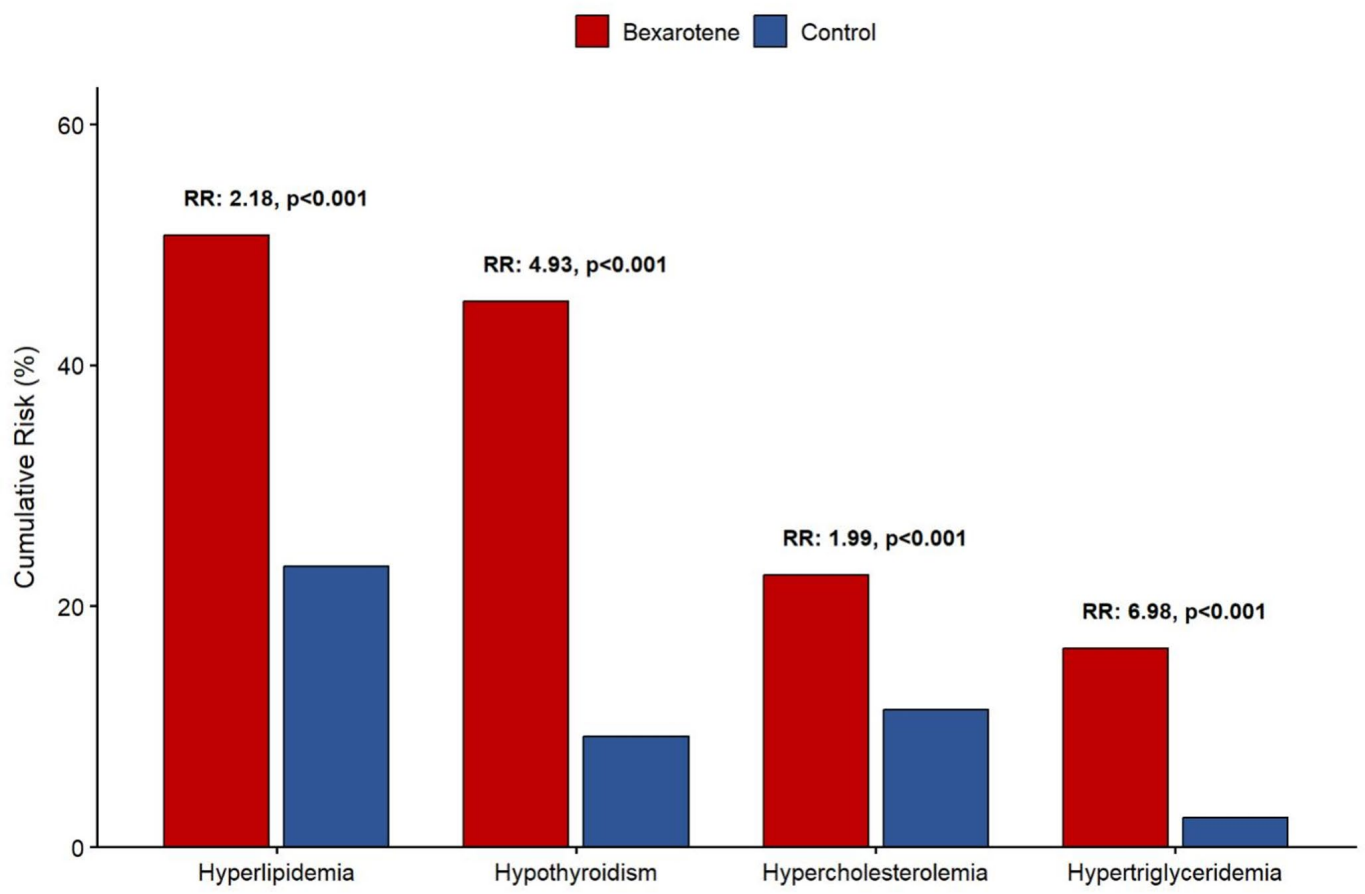
Cumulative risk of abnormal metabolic and lipid laboratory findings. Note: RR= relative risk.

Figure 1 portrays the cumulative risks of abnormal metabolic and lipid laboratory findings between the bexarotene and control cohorts.

## Cardiovascular-related events

### Acute Myocardial Infarction (AMI)

A total of 68 and 73 AMI events were recorded during follow-up between those treated with bexarotene and those not, respectively. No statistically significant differences were noted in the cumulative risk (3.1% vs. 3.3%, RR = 0.92, 95% CI: 0.67–1.28, *p* = 0.631) or time-dependent risk of AMI (HR = 0.95, 95% CI: 0.68–1.31, *p* = 0.730) between the two groups (Supplementary Fig. 6). The proportional hazards assumption was not violated (*p* = 0.453).

### Cerebral Infarction (Stroke)

A total of 63 and 73 stroke events were observed during follow-up between the bexarotene and no bexarotene cohorts, respectively. No statistically significant differences were noted in the cumulative risk (2.9% vs. 3.4%, RR = 0.85, 95% CI: 0.61–1.19, *p* = 0.344) or time-dependent risk of stroke (HR = 0.90, 95% CI: 0.62–1.24, *p* = 0.514) between the two groups (Supplementary Fig. 7). The proportional hazards assumption was not violated (*p* = 0.713).

### Heart Failure (HF)

A total of 176 and 187 diagnoses of HF were documented during follow-up between those treated with bexarotene and those not, respectively. The cumulative risk (8.1% vs. 8.8%, RR = 0.92, 95% CI: 0.76–1.12, *p* = 0.408) and the time-dependent risk of HF (HR = 0.93, 95%CI: 0.75–1.14, *p* = 0.455) were identical between the two groups (Supplementary Fig. 8). The proportional hazards assumption was not violated (*p* = 0.357).

### Adapted-MACE (AMI – Stroke – HF – Revascularization)

**Fig. 2:**
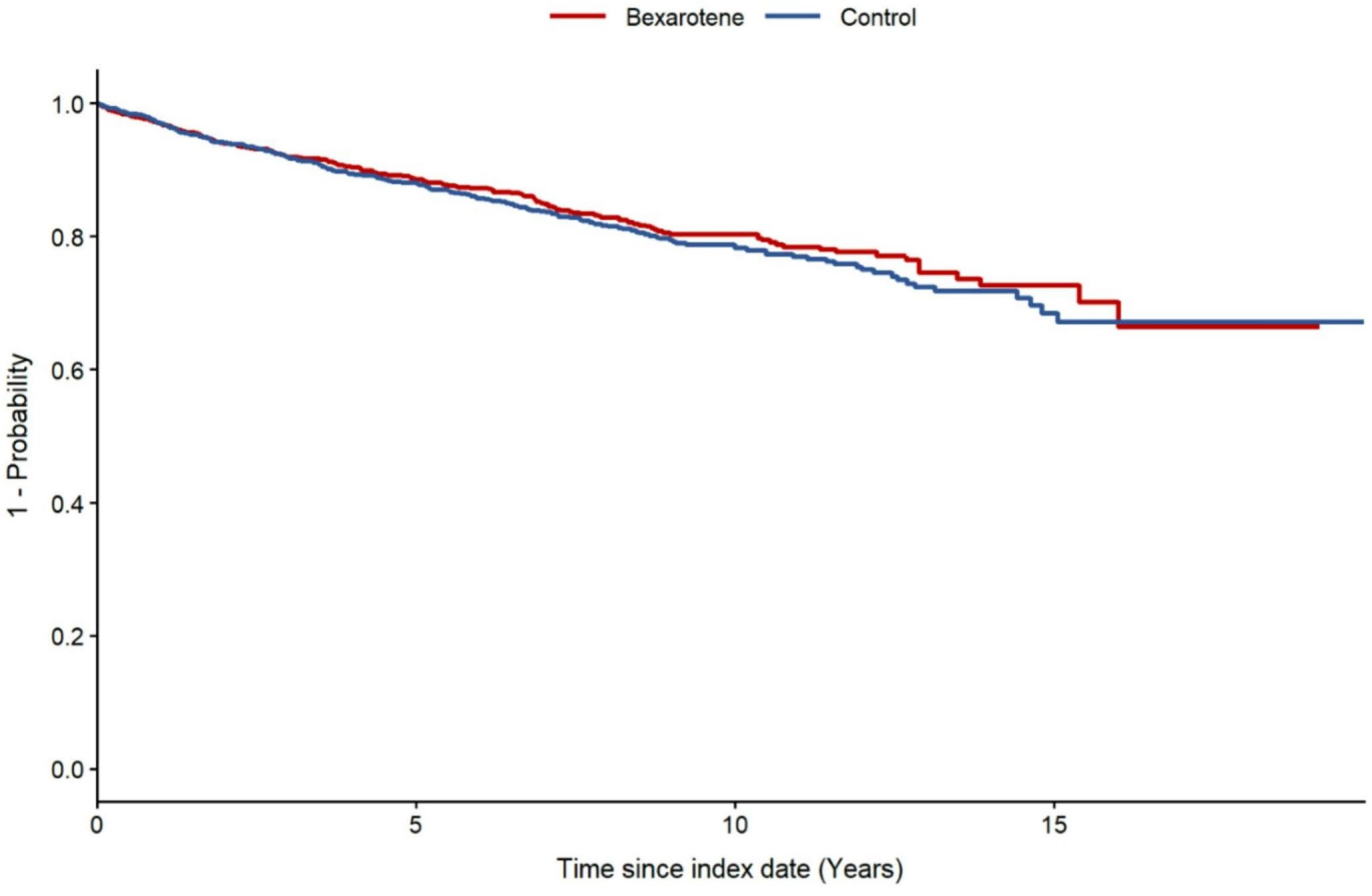
Kaplan Meier plot for the adapted-MACE

The composite adapted outcome of MACE in our study was defined as an event of AMI, stroke or HF, combined with revascularization procedures. A total of 243 and 253 events were observed during follow-up in the bexarotene and no bexarotene cohorts, respectively. The cumulative risk was 11.5% and 12.4%, respectively (RR = 0.92, 95% CI: 0.78–1.09, *p* = 0.350). No differences were noted in the time-to-event analysis (HR = 0.93, 95% CI: 0.78–1.11, *p* = 0.425) (Fig. 2). The proportional hazards assumption was not violated (*p* = 0.687).

**Fig. 3:**
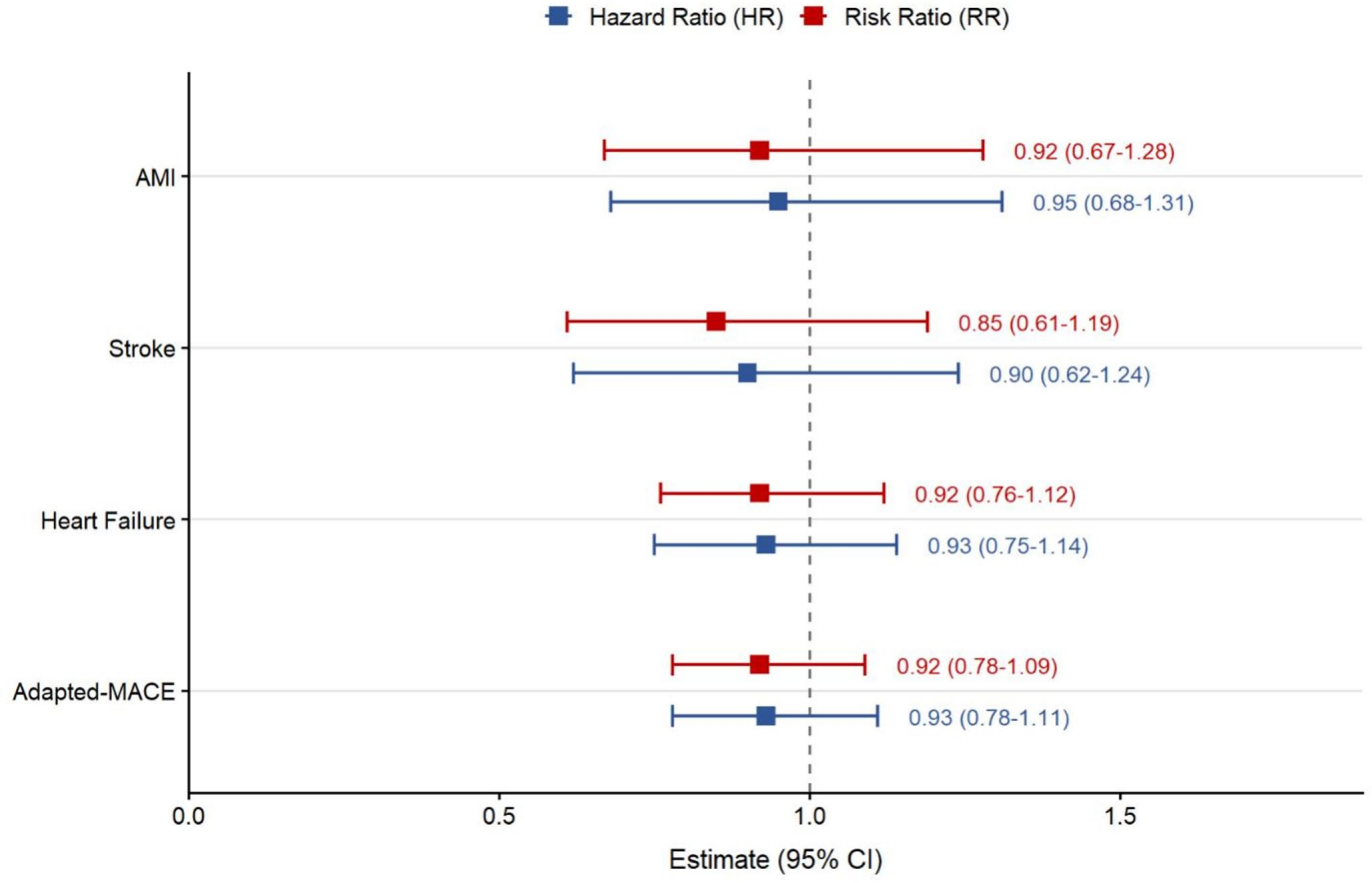
Forest plot of effect estimates for cardiovascular outcomes. *Note*: *RR* or *HR* > 1 denote increased risk of cardiovascular outcomes with Bexarotene, *AMI* Acute myocardial infarction, *MACE* Major adverse cardiovascular event, *RR* relative risk, *HR* hazard ratio, *CI* confidence interval

Moreover, Fig. 3 presents the aforementioned effect estimates for all of the outcomes studied.

### Post-index rates of antilipemic and thyroid medication use

#### Antilipemic agents

Excluding patients who were prescribed antilipemic agents prior to the index date, a total of 1,070 patients in the bexarotene arm and 1,430 patients in the control arm had available data for subsequent prescriptions. Among them, 507 (47.4%) and 330 (23.1%) individuals required therapy with antilipemic agents (OR = 3.00, 95% CI: 2.53–3.57, *p* < 0.001).

### Thyroid modifiers

Excluding patients who were prescribed thyroid modifiers prior to the index date, a total of 1,455 patients in the bexarotene arm and 1,916 patients in the control arm had available data for subsequent prescriptions. Among them, 600 (41.2%) and 106 (5.5%) required therapy with thyroid modifiers (OR = 11.98, 95% CI: 9.60-14.96, *p* < 0.001).

## Discussion

This study represents the largest real-world analysis to date evaluating the long-term cardiovascular safety of bexarotene in patients with MF. Our findings demonstrate that while bexarotene is associated with a profound increase in metabolic and endocrine derangements, these abnormalities did not seem to translate into an increased risk of major adverse cardiovascular events or heart failure over a median follow-up of 4.0 years.

The significant increase in metabolic lab abnormalities observed in our cohort is consistent with the known pharmacodynamics of selective RXR agonists [17]. RXR agonists suppress the thyroid-stimulating hormone (TSH) β-subunit gene, leading to rapid central hypothyroidism, while simultaneously inducing dyslipidemia through increased VLDL production and decreased CETP-mediated clearance [18]. In the general population, such severe elevations in triglycerides and LDL are established precursors to accelerated atherosclerosis and MACE [13, 19]. However, our data suggest that the cardiovascular impact of bexarotene’s metabolic burden may be mitigated in clinical practice.

Several factors may explain the lack of observed cardiovascular complications despite severe metabolic signals. First, bexarotene-induced hyperlipidemia and hypothyroidism are typically managed with aggressive, concurrent pharmacotherapy, such as statins, fibrates, and levothyroxine, as part of standard-of-care protocols [20, 21]. In this context, we observed a massive 12-fold increase in the initiation of thyroid modifiers and a 3-fold increase in new antilipemic prescriptions following bexarotene exposure in our study. Additionally, our baseline data show that a notable proportion of patients were on relevant medications at index, a figure likely to increase during the treatment course, per abnormal laboratory findings [22, 23]. Second, these derangements occurring only during active therapy, may not provide a sufficient cumulative exposure time to drive the chronic process of plaque rupture and acute ischemic events within the follow-up window [24–26]. Our data suggest that this treatment-related metabolic stress may be insufficient to overcome the baseline vascular stability of patients, particularly when mitigated by standard-of-care prophylaxis or interventions.

Finally, the median follow-up window of approximately 4.0 years may be considered short relative to the traditional, multi-year timeline required for chronic dyslipidemia to manifest as clinically evident, plaque-driven atherosclerosis. In the general population, the progression from the onset of metabolic risk factors to acute MACE is typically a decadal process [27, 28]. However, capturing extended longitudinal exposure in real-world CTCL datasets is challenging due to high rates of treatment discontinuation. For instance, a nationwide post-marketing surveillance study of bexarotene demonstrated that 41.2% of Japanese CTCL patients discontinued bexarotene within a median observation framework of just 9.1 months due to adverse events or disease progression [29].

The primary strengths of this study lie in its scale and novelty. To our knowledge, this is the first large-scale real-world study evaluating the risk of bexarotene-induced cardiovascular outcomes in MF. By utilizing the TriNetX Global Health Research Network, we were able to overcome the inherent limitations of previously published small-scale case series. Furthermore, the application of rigorous 1:1 PSM achieved near-perfect balance across a comprehensive array of baseline covariates, including pre-existing lipid profiles and comorbidities, blood pressure, and cardiovascular medications. This methodological rigor potentially isolated bexarotene exposure as the primary driver of the observed metabolic outcomes while mitigating selection bias.

The inclusion of baseline lipid profiles, blood pressure parameters, and corresponding cardiovascular medications within the PSM model could theoretically risk overmatching if these variables functioned as intermediate factors along the true causal pathway. However, adjusting for these pre-index metabolic characteristics was essential to isolate the specific impact of bexarotene from baseline confounding by indication, given that patients channeled toward systemic retinoid therapy in real-world clinical settings frequently harbor inherently higher baseline cardiovascular risks than those managed with skin-directed or less toxic systemic options. While this conservative framework prioritizes internal validity by eliminating pre-existing cardiovascular imbalances, it ensures that the observed post-index metabolic shifts can be independently evaluated relative to subsequent cardiovascular events.

Nevertheless, several limitations must be considered when interpreting these results. First, as a retrospective registry-based study utilizing electronic health records, our analysis is dependent on the accuracy of ICD-10 coding and the consistency of reporting across various institutions. Second, despite rigorous and extensive matching, some of the covariates were not available for the total population; an inherent limitation of this study design. Third, the nature of bexarotene-induced metabolic shifts means that patients may have had varying durations of exposure, and our follow-up window may be insufficient to capture the long-term sequelae of lipid-driven atherosclerosis. Moreover, although TriNetX provides a global sample, the results may still be influenced by regional variations in the standard-of-care management of bexarotene-induced side effects.

Notably, the nature of the database limited our ability to explicitly track temporary treatment interruptions or permanent discontinuations, which represents a potential source of exposure misclassification bias. In real-world clinical practice, management of CTCL with oral retinoids often involves a circular model of treatment adjustment where clinician-directed stops or dose adjustments are frequently triggered by unacceptable adverse profiles [30]. Recent literature highlights that bexarotene carries a significantly higher risk of toxicity-driven intervention than other systemic retinoids, such as acitretin, with patients requiring therapeutic switches primarily due to severe blood lipid anomalies and metabolic derangements [31]. By failing to account for these treatment interruptions or discontinuations, our findings may be limited by an inherent misclassification or exposure bias.

Additionally, the markedly increased incidence of hypothyroidism and dyslipidemia observed in the treatment group may be partially influenced by ascertainment bias, as patients initiating bexarotene typically undergo more intensive, guideline-mandated laboratory surveillance compared to controls, which naturally increases the likelihood of event detection rather than reflecting a pure biological effect. Moreover, although we adjusted for biological surrogates of tumor burden (e.g. elevated LDH, lymphocytosis, eosinophilia, nodal involvement, Sezary disease [16]), granular clinical data such as the type and extent of skin lesions, TNMB staging, prior treatment modalities and hospitalization burden were not available, which may limit the assessment of disease-specific confounding. Finally, missing data were handled via the platform’s native presence-absence operational logic rather than mathematical imputation, which may introduce residual confounding from unrecorded parameters.

## Conclusion

In conclusion, this study provides the largest real-world evaluation to date of cardiovascular outcomes among patients with MF treated with bexarotene. While our findings are hypothesis-generating and should be interpreted within the limitations of an observational database study, they suggest that despite a substantial increase in metabolic and endocrine abnormalities, bexarotene treatment was not associated with an increased risk of adverse cardiovascular events during the observed follow-up period. These findings provide reassurance regarding the short- to intermediate-term cardiovascular outcomes of bexarotene-treated patients when appropriate monitoring and management of lipid and thyroid abnormalities are maintained. Future prospective studies with longer follow-up are warranted to clarify the long-term vascular consequences of these treatment-related metabolic changes.

## Supplementary Information

Below is the link to the electronic supplementary material.

Supplementary Material 1

## Data Availability

The data analyzed was accessed via the TriNetX global research network platform and is available there.
