# Supplementary material for: Real-world cardiovascular safety profile of bexarotene for the treatment of mycosis fungoides
Source: Clin Exp Med. 2026 Aug 10;26(1):295. doi: 10.1007/s10238-026-02282-5 (PMC13457487; doi:10.1007/s10238-026-02282-5)
Supplement: Supplementary file 1 [file 10238_2026_2282_MOESM1_ESM.docx]

**Real-World Cardiovascular Safety Profile of Bexarotene for the Treatment of Mycosis Fungoides**

**Supplementary Material**

**Supplementary Figure 1.** Propensity score density graphs, before (left) and after matching (right)

**Note:** Purple= Bexarotene, Green= Control

**Supplementary Figure 2.** Kaplan Meier plot for hypertriglyceridemia events

**Note:** Purple= Bexarotene, Green= Control

**Supplementary Figure 3.** Kaplan Meier plot for hypothyroidism events

**Note:** Purple= Bexarotene, Green= Control

**Supplementary Figure 4.** Kaplan Meier plot for hypercholesterolemia events

**Note:** Purple= Bexarotene, Green= Control

**Supplementary Figure 5.** Kaplan Meier plot for hyperlipidemia events

**Note:** Purple= Bexarotene, Green= Control

**Supplementary Figure 6.** Kaplan Meier plot for Acute Myocardial Infarction

**Note:** Purple= Bexarotene, Green= Control

**Supplementary Figure 7.** Kaplan Meier plot for Cerebral Infarction (stroke)

**Note:** Purple= Bexarotene, Green= Control

**Supplementary Figure 8.** Kaplan Meier plot for Heart Failure

**Note:** Purple= Bexarotene, Green= Control
